# IsoBED: a tool for automatic calculation of biologically equivalent fractionation schedules in radiotherapy using IMRT with a simultaneous integrated boost (SIB) technique

**DOI:** 10.1186/1756-9966-30-52

**Published:** 2011-05-09

**Authors:** Vicente Bruzzaniti, Armando Abate, Massimo Pedrini, Marcello Benassi, Lidia Strigari

**Affiliations:** 1 Laboratory of Medical Physics and Expert System, Regina Elena Cancer Institute, Via E. Chianesi 53, 00144, Rome, Italy

## Abstract

**Background:**

An advantage of the Intensity Modulated Radiotherapy (IMRT) technique is the feasibility to deliver different therapeutic dose levels to PTVs in a single treatment session using the Simultaneous Integrated Boost (SIB) technique. The paper aims to describe an automated tool to calculate the dose to be delivered with the SIB-IMRT technique in different anatomical regions that have the same Biological Equivalent Dose (BED), i.e. IsoBED, compared to the standard fractionation.

**Methods:**

Based on the Linear Quadratic Model (LQM), we developed software that allows treatment schedules, biologically equivalent to standard fractionations, to be calculated. The main radiobiological parameters from literature are included in a database inside the software, which can be updated according to the clinical experience of each Institute. In particular, the BED to each target volume will be computed based on the alpha/beta ratio, total dose and the dose per fraction (generally 2 Gy for a standard fractionation). Then, after selecting the reference target, i.e. the PTV that controls the fractionation, a new total dose and dose per fraction providing the same isoBED will be calculated for each target volume.

**Results:**

The IsoBED Software developed allows: 1) the calculation of new IsoBED treatment schedules derived from standard prescriptions and based on LQM, 2) the conversion of the dose-volume histograms (DVHs) for each Target and OAR to a nominal standard dose at 2Gy per fraction in order to be shown together with the DV-constraints from literature, based on the LQM and radiobiological parameters, and 3) the calculation of Tumor Control Probability (TCP) and Normal Tissue Complication Probability (NTCP) curve versus the prescribed dose to the reference target.

## Background

Irradiation techniques with Intensity Modulated Radiotherapy (IMRT) allow doses to be delivered to the target with a high conformation of prescribed isodose, sparing Organs at Risk (OARs), compared to conventional 3D-CRT techniques. Another advantage of the IMRT technique is the possibility to achieve the so-called Simultaneous Integrated Boost (SIB), which provides different levels of therapeutic doses to different target volumes during the same treatment session, once the fraction number has been set [1-5].

Historically, to obtain the desired tumor control, the doses were determined using a conventional fractionation that ranged between 50 to 70 Gy at 2 Gy per fraction.

Whereas, in order to obtain Tumor Control Probability (TCP), equivalent to that of a conventional fractionation, the total dose simultaneously delivered to the targets have to be determined according to the Linear Quadratic Model (LQM) to be used with the SIB technique [6]. Thus, the dose per fraction to PTVs and/or boost may differ by 2 Gy per fraction.

Based on the Biological Equivalent Dose (BED) formalism, a new total dose and the fraction dose can be calculated in order to obtain the same biological effect, named IsoBED herein [7,8].

The paper aims to: 1) describe home-made software, based on the IsoBED formula, able to calculate the total dose and the dose per fraction with the same TCP as the conventional fractionation, that will be used with the SIB technique, 2) import the DVHs from different TPSs or different plans, convert them into a normalized 2 Gy-fraction-Volume Histogram (NTD_2_-VH) and compare these amongst themselves and with the Dose-Volume constraints (DV- constraints), 3) calculate and compare the TCPs and the Normal Tissue Complication Probabilities (NTCPs) obtained from different DVHs.

## Methods

### Radiobiological formulation

This approach was based on the LQM, widely used for fractionated external beam-RT, to describe the surviving fraction (*sf) *of cells in the tissues exposed to a total radiation dose D (expressed in Gy) and to a dose per fraction *d*(expressed in Gy). The logarithm of the surviving fraction, in the absence of any concurrent re-population, can be expressed as:(1)

Where *α *is a radiobiological parameter, the BED was defined as:(2)

and the (*α*/*β*) ratio is a parameter which takes into account the radiobiological effect of fractionation in tumor or OARs.

Equation (2) is the basis on which a comparison of different treatment strategies is performed.

In order to obtain the same cell survival with two fractionations having a total dose (D_1 _and D_2_) and dose per fraction (d_1 _and d_2_), the following equation can be invoked:(3)

i.e.(4)

and expressed in terms of number of fractions *n*_*1 *_and *n*_*2 *_respectively(5)

If we have a fractionation schedule with *BED*_1 _characterized by D_1_, d_1 _and n_1 _and a new schedule is required, in terms of n_2 _and d_2_, with the same *BED*_1_, then, substituting n_2 _by *n *in equation (5) we obtain:

i.e.

and then(6)

The solution of which is:(7)

Where d_2 _is the new dose per fraction delivered in *n *fractions, resulting in a new total dose D_2 _= d_2 _*n*,

Equation (7) is valid for both PTVs and OARs (following the LQM).

### The IsoBED software

The software has been developed using the Microsoft Visual Basic 6.0. The main form - the IsoBED Calculator- gives a choice between IsoBED calculation and DVHs analysis modules.

#### IsoBED Calculation

The software allows the anatomical district to be selected. The user has to introduce the total dose, dose per fraction (generally 2 Gy per fraction) for each target (up to 3) and, the (*α*/*β*) ratio of investigated tumor must be inserted to calculate the corresponding BED.

Then the software requires the selection of the reference target (which determines the fractions number in the SIB treatment), in order to calculate the new fractionation for the remaining targets, based on equation (7). Furthermore, the software permits a comparison of the biologically equivalent schedules using hyper/hypo-fractionated as well as conventional regimes. It also includes a database with the main DV- constraints at 2 Gy per fraction for different OARs derived from literature and clinical experience in the radiotherapy department of our Institute [9-20] which may be upgraded by the user.

The DV-constraints are converted to those of the new schedule (i.e. hypo or hyper-fractionated) calculated by IsoBED.

Then the converted constraints for OARs can be printed and used as constraints for IMRT optimization.

#### DVH import and radiobiological analysis

After the IMRT optimization using commercial TPSs (such as: BrainScan, Eclipse, Pinnacle), the obtained DVHs can be imported to our software and can be used to compare techniques and/or dose distributions from the same or different TPSs.

The software automatically recognizes the DVH file format exported from each TPS source and imports it into the patient directory without any changes. In particular, import procedures consist of copying DVH files into a subfolder with the patient's name, contained in a directory where the IsoBED.exe file is held.

Then, a specific window permits the analysis of DVHs to be carried-out. Cumulative or differential DVHs can be visualized after setting dose per fraction and fraction number. In this window up to five plans imported from BrainScan, Eclipse and Pinnacle can be compared. The volumes and the minimum, mean, median, modal and maximum doses can be visualized for OARs and PTVs.

For each volume the software calculates NTD_2_VH (Appendix 1 equation 1.6) by using the appropriate (*α*/*β*)ratio, which may be changed by the user.

Finally, the TCP, NTCP and Therapeutic Gain (P+) curves can be calculated from the DVHs based on radiobiological parameter sets, derived from literature but upgraded by the user, according to the formulas reported in Appendix 1 [21-27].

To illustrate this user friendly IsoBED software some case examples are shown.

### Example cases

The following test cases were considered in order to illustrate the usefulness of the home made software for comparing sequential versus SIB plans for three clinical treatments in this paper.

#### Prostate Case

The first case regards irradiation using IMRT of prostate and pelvic lymph nodes.

The comparison was made between the sum of 2 sequential IMRT plans (50 Gy to the lymph nodes and prostate at 2 Gy per fraction followed by another 30 Gy at 2 Gy per fraction only on the prostate for a total of 40 fractions) and an SIB IMRT plan [7].

Assuming the same fractionation for prostate, the total dose and dose per fraction of pelvic lymph nodes were calculated with the IsoBED software, using an (*α*/*β*)ratio = 1.5 Gy for both targets [28,29].

The treatment plans were developed using Helios module of Eclipse TPS (Varian Medical System). All 3 treatment plans were performed with the same geometry using 5 coplanar fields (angles: 0, 75, 135, 225 and 285 degrees) with the patient in prone position.

**Figure 1:**
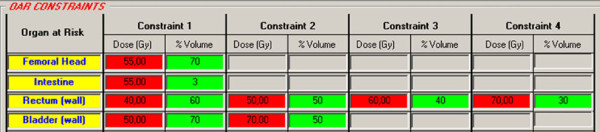
**OAR DV-constraints provided by IsoBED for prostate case**.

The primary plan acceptance criteria should meet treatment goals (prescribed dose to >95% of the volumes) for all target while keeping the rectum, bladder, femoral heads and intestine dose under the DV-constraints provided by software for sequential versus SIB plans (Figure 1) [10-12].

#### Head and Neck Case

The second case regards the treatment of a rinopharynx cancer patient.

The prescribed dose was 53 Gy at 2.12 Gy per fraction to the Planning Elective Tumor Volume (PETV, i.e. PTV54), 59.36 Gy at 2.12 Gy per fraction to the Planning Clinical Target Volume (PCTV, i.e. PTV60) and 69.96 Gy at 2.12 Gy per fraction to the Planning Gross Target Volume (PGTV, i.e. PTV70).

The first plan, the sequential treatment, was calculated to deliver 53 Gy in 25 fractions to PETV followed by 6.36 Gy in 3 fractions to the PCTV and another 10.6 Gy in 5 fractions to the PGTV, for a total of 33 fractions.

For the SIB plan, the IsoBED doses derived from prescription and the calculated doses from our software were considered in order to deliver 69.96 Gy in 33 fractions to the PGTV.

**Figure 2:**
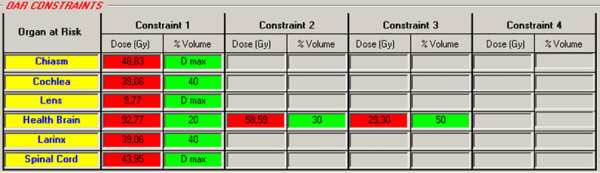
**OAR DV-constraints provided by IsoBED for Head & Neck case**.

The setup of the IMRT plan was calculated with Pinnacle 8.0 m TPS (Philips Medical Systems, Madison, WI) and based on seven 6 MV photon beam techniques (angles 35, 70, 130, 180, 230, 290 and 330 degrees) [13]. The acceptance criteria of the primary plan had to meet treatment goals (prescribed dose to >95% of the volumes) for all target while keeping the dose of the spinal cord, brain-stem, optic structures (optic nerves, chiasm and lens) and larynx under DV-constrains of sequential and SIB plans (Figure 2). For parotids the mean doses were considered under 32 Gy [14-17].

#### Lung case

In a lung cancer patient two volumes had to be irradiated in a hypofractionaction regime [18]. The prescription of the sequential technique was: PTV to receive 40 Gy at 10 Gy per fraction and for the boost an additional fraction of 10 Gy. The SIB technique consisted of an IMRT plan, for which the dose were calculated by IsoBED software, so that the boost received 50 Gy in 5 fractions.

**Figure 3:**
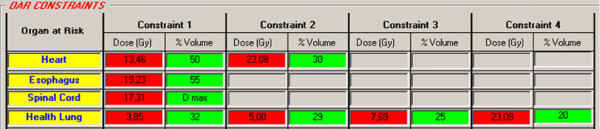
**OAR DV-constraints provided by IsoBED for Lung case**.

In both cases, the plans were performed by the Pinnacle TPS using 6 MV photon energy and 3 coplanar fields (angles 20, 100 and 180 degrees). The acceptance criteria for the primary plan had to meet treatment goals (prescribed dose to >95% of the volumes) for all target while keeping the maximum dose of the healthy lung, spinal cord, esophagus and heart under DV-constrains of sequential and SIB plans (Figure 3) [19,20].

### Data analysis

The plan sum was created from the sequential IMRT plans which had to be compared with the IMRT SIB plan. All plans were exported from TPSs and imported into the IsoBED software to calculate and compare NTD_2_VH, TCP, NTCP and P+.

## Results

### IsoBED Calculation

**Figure 4:**
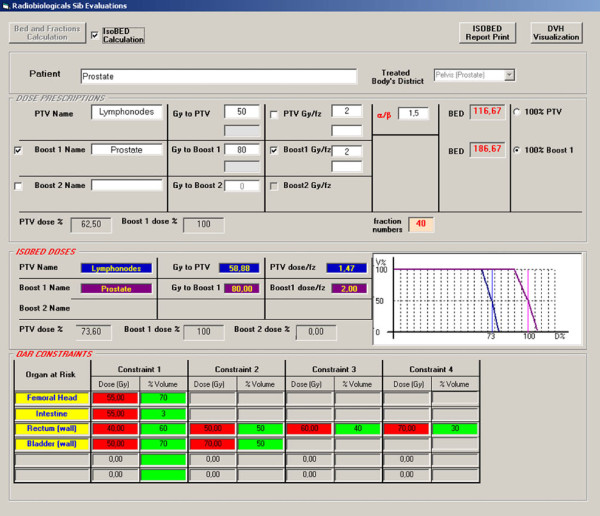
**Example of IsoBED calculation for the case of prostate and lymph nodes treatment**.

Figure 4 shows an example of IsoBED calculation for the case of prostate cancer and lymph node treatment. The screen is constituted by an area denominated "DOSE PRESCRIPTION" where the dose prescriptions desired for each PTV and (*α*/*β*)value are inserted. For the BED calculation it is necessary, as previously described, to select the target, named reference target, that will determine the fraction number. Thus, BED values are calculated by clicking on the button "BED and Fractionaction Calculation".

Then the SIB schedule is calculated by selecting the control box "IsoBED Calculation". The results of such evaluations are visualized in the "IsoBED DOSES" area. The dose limits are visualized in the "OAR CONSTRAINTS" area.

### DVH import

Import procedures consist of copying DVH files, exported from TPS, in a folder with the patient's name contained in a directory where an IsoBED.exe file is installed. DVH files are different depending on the TPS source. IsoBED can import DHV data files from Eclipse, Pinnacle and Brainscan.

### Dose distribution and radiobiological analysis

**Figure 5:**
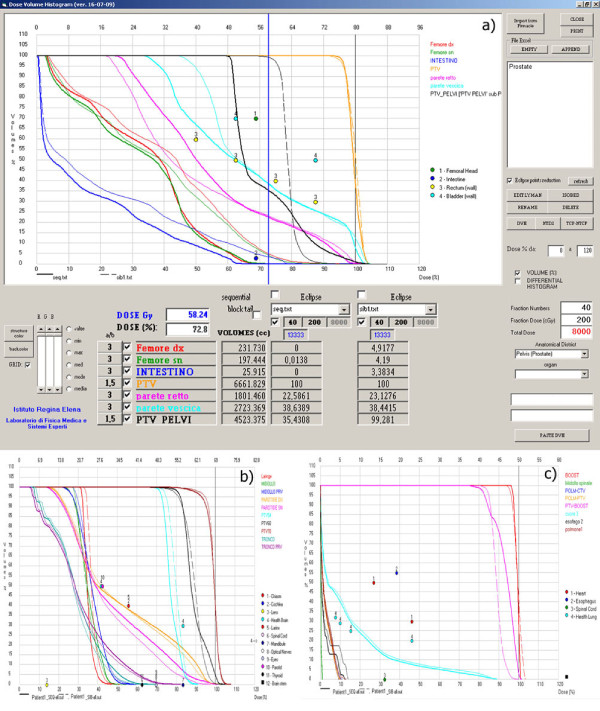
**DVHs imported from TPSs for Sequential and SIB Technique in a) prostate, b) Head & Neck and c) Lung cases**. Numered circles represents the OAR costraints.

**Figure 6:**
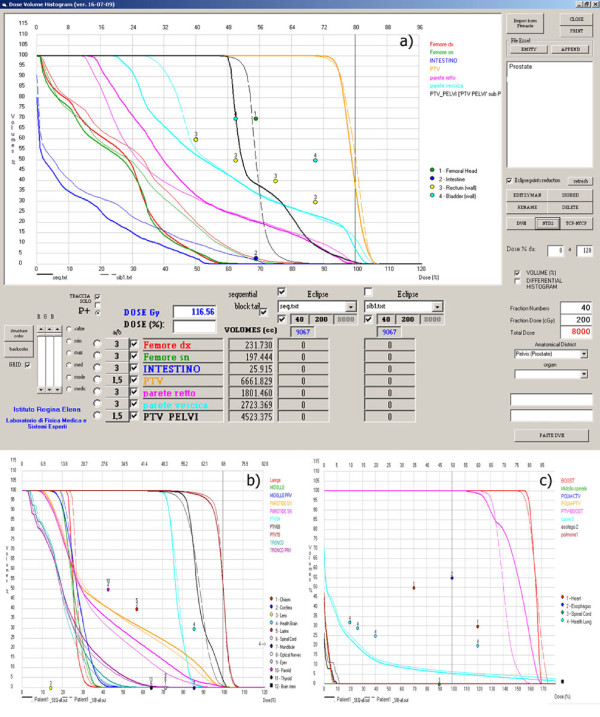
**NTD_2_-VH for Sequential and SIB Technique in a) prostate, b) Head & Neck and c) Lung cases**. Numered circles represents the OAR costraints.

**Figure 7:**
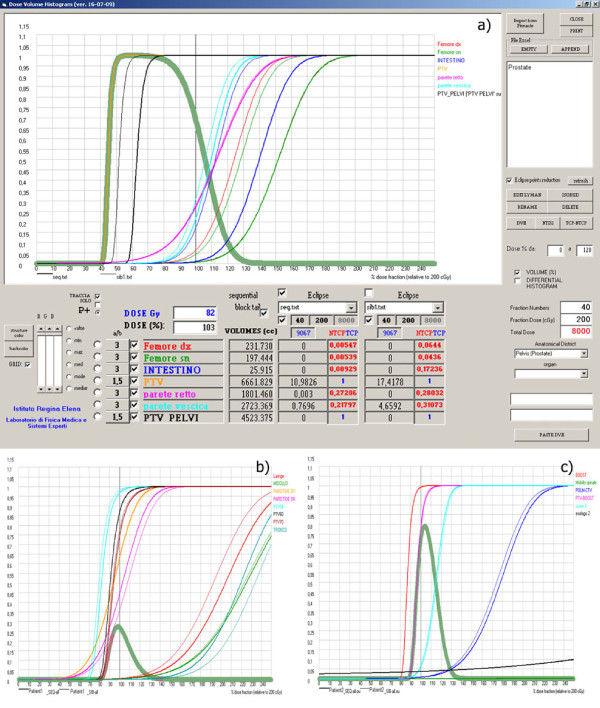
**Radiobiological curves (TCP, NTCP and P_+_) for Sequential and SIB Technique in a) prostate, b) Head & Neck and c) Lung cases**.

Figures 5, 6 and 7 show different screens generated by the software through which different types of evaluations for prostate-pelvis, head & neck and lung cases can be performed. On the right side of the screen there is a window where the patient of interest can be selected, while in the lower part of the screen the fraction number, dose per fraction and the district of interest can be set. Thus, the total dose can be calculated and all the imported DVHs are visualized.

Figures 5a, 5b and 5c show the DVHs imported from TPSs calculated with different modalities (SIB and sequential). The user can choose which volume of interest to view by selecting them from a list visualized at the lower-left corner of the screen. Furthermore, in the same area, the total volume or one between, the minimum, maximum, average, median and modal dose percentage for each plan and each structure shown in the histogram is displayed.

In order to perform radiobiological calculations the (*α*/*β*)values can be set for each structure by choosing a dropdown menu in which the list of parameters incorporated in a dedicated database appears. These values are derived from literature data and from experience at our Institute [9-20]. The "NTD2" button transforms every DVH into the NTD_2_VH (Figures 6a, 6b and 6c).

Finally, the TCP, NTCP and P+ curves against the dose prescribed to the reference target can be calculated with the "TCP-NTCP" button and their values are shown in the lower area of the screen (Figures 7a, 7b and 7c).

### Software Validation

All the outcomes from IsoBED software were compared with an automatic excel spreadsheet specially designed for this purpose. In particular, the outcomes from IsoBED calculation and from DVH import and radiobiological analysis modules were tested. The results obtained from the comparison made it possible to validate the software.

## Discussion

The introduction of the IMRT technique in clinical practice, including the SIB approach, requires new treatment schedules able to guarantee the same BED of conventional fractionations to be drawn up. Automatic software that does this is a useful tool when making these estimates, particularly with regard to evaluations and for comparing different forms of DVHs and radiobiological parameters [30-35].

The software, described in this paper, is based on the BED calculation and on LQM. Unlike other software, it allows fractionation schedules to be calculated in SIB-IMRT treatment techniques with both conventional and hypo-fractionation regimes, after setting the desired dose per fraction.

Similar to Bioplan [30], the IsoBED software is an analysis tool used to compare DVHs with different TPSs or different irradiation techniques.

In addition, this software allows a comparison between plans using NTD2VH. This is a very interesting and useful aspect as it is possible to take into consideration simultaneously the end-points of different OARs.

Moreover, the import of DVHs enables dosimetric and radiobiological comparisons between different TPSs, which is an important issue because this may be used as quality control for treatment planning systems when simple geometry of phantoms are assumed [36,37].

In addition, the TCP and NTCP curves can be calculated to select the best treatment plans to be discussed with physicians. In fact, the P+ curve can be used to confirm the dose prescription to reference target. In particular, the maximum peak of the P+ curve indicates the dose per fraction to reference target giving the maximum TCP value with the lowest combination of NTCPs.

Furthermore, the possibility of changing the (*α*/*β*)value while designing the fractionation scheme might aid the prediction of different effects (such as acute and late effect) related to clinical trials.

Finally, the possibility of updating the radiobiological parameters for OARs stored in the internal database permits us to take into consideration the proven clinical experience of users. The software calculates the radiobiological DV-constrains for different fractionations as shown in the case examples (Figure 1, 2 and 3).

An issue to be considered regards the use of the LQM adopted by IsoBED. In fact, this model is strictly applicable with intermediate doses while its applicability with doses higher than 18-20 Gy per fraction is under debate [38,39]. Nevertheless, the use of simple analytic models may provide useful suggestions in clinical radiotherapy.

## Conclusions

IsoBED software based on LQM allows one to design treatment schedules by using the SIB approach, importing DVHs from different TPSs for dosimetric and radiobiological comparison. It also allows to select and evaluate the best approach able to guarantee maximum TCP and at the same time the minimum NTCP to the organs at risk.

## Competing interests

The authors declare that they have no competing interests.

## Authors' contributions

Conception and design: VB, MB and LS. Development of software: VB and MP. Analysis and interpretation of the data using IsoBED: AA, LS, MP and VB. Drafting of the manuscript: VB, AA, MB and LS. Final approval of the article: All authors read and approved the final manuscript.

## Appendix 1

### TCP

Assuming that the cell survival in a tumor follows a binomial statistic, the requirement of total eradication of all clonogenic cells yields the Poisson formula for TCP:(1.1)

where N* is the total initial number of tumor clonogenic cells and *sf *is the surviving fraction.

### NTCP model

The Lyman-Burman Kutcher (LBK) model was used to calculate the NTCP. For uniform irradiation of a fraction *v*_*eff *_of the organ at a maximum dose at 2 Gy per fraction, *NTD*_2,MAX_, the NTCP can be calculated by:(1.2)

where s is defined as:(1.3)

where m and *TD*_50 _(*v*_*eff*_) are the slope of the NTCP curve versus the dose and the tolerance dose at 2 Gy per fraction to a fraction *v*_*eff *_of the organ, respectively.

### DVH reduction

In order to generalize the LBK method each DVH has been converted into a single value using a DVH reduction method.

The effective volume (*v*_eff_) method was chosen as a histogram reduction scheme for non-uniform organ irradiation:(1.4)

where *D*_i _is the dose delivered to the volume fraction *v*_*i*_, K is the number of points of the differential DVH, *D*_*max *_is the maximum dose and n is a parameter related to organ response to radiation (n = 0,1 for serial and parallel organs, respectively). By Eq. (1.4), an inhomogeneous dose distribution is converted into an equivalent uniform irradiation of a fraction *v*_*eff *_of the organ treated at the maximum dose (*D*_*max*_).

The *TD*_50 _(*v*_*eff*_) can be calculated using the following equation:(1.5)

where *TD*_50_(1) is the tolerance dose to the whole organ, leading to a 50% complication probability.

In order to take into account the new dose per fraction (d_i _= *D*_*i*_/N and d = *D*_*max*_/N, where N is the number of fractions), both *D*_*i*_(received by the volume fraction *v*_*i*_) and the maximum dose *D*_*max *_are converted to the nominal standard dose (i.e. *NTD*_2 _= {*NTD*_2, *i*_}), applying the following equations:(1.6)

and(1.7)

respectively.

Equation (1.4) becomes:(1.8)

By using this formula, each dose step in the DVHs was corrected separately. This formalism presumes complete cellular repair between treatment fractions and neglects the role of cellular re-population. The latter assumption is valid for late-responding normal tissues but is inaccurate for acute-responding tissues and tumors. This limitation may be important when using the LQM to compare treatment schedules differing in overall treatment times in terms of their acute effects (for which time-dependent repopulation may be important). For late effects, time factors are generally thought to be of minor importance.

### Therapeutic Gain

Therapeutic gain is used to compare optimization outcomes in treatment plans calculated with different modalities taking into account both tumor control and normal tissue complications. The following expression is used:(1.9)
